# Effect of Dexmedetomidine with or without Midazolam during procedural dental sedation in children: a randomized controlled clinical trial

**DOI:** 10.1186/s12903-024-04992-2

**Published:** 2024-10-26

**Authors:** Amira A. ElKhatib, Tamer A. M. Ghoneim, Karin M. L. Dowidar, Nadia A. Wahba

**Affiliations:** 1 Faculty of Oral and Dental Medicine, Kafrelsheikh University, Kafr El Sheikh, Egypt; 2 Faculty of Medicine, Alexandria University, Alexandria, Egypt; 3 Faculty of Dentistry, Alexandria University, Alexandria, Egypt

**Keywords:** Dental sedation, Dexmedometidine, Midazolam, Nebulizer, Preschoolers

## Abstract

**Background:**

Dental anxiety is a global problem in the realm of pediatric dentistry. The use of procedural sedation is recommended to avoid substandard or unsafe dental treatment in preschoolers. This study aimed to compare the effect sedation with Dexmedetomidine with or without Midazolam in terms of sedation level, analgesic effect and ease of treatment completion in preschool children.

**Methods:**

A triple blind randomized controlled clinical trial comprised 72 healthy uncooperative children, 4–6 years old, were randomly allocated into three groups of 24 patients each: Group I patients were sedated with nebulized 5 μg/kg Dexmedetomidine (DEX), Group II with nebulized 3 μg/kg DEX followed by nebulized 0.3 mg/kg Midazolam (MID), and Group III with nebulized 0.5 mg/kg MID. Along the session, the three regimens were assessed and compared during the sedation level (children responsiveness) using the Modified Observer’s Assessment of Alertness/Sedation Scale, the analgesic effect using the Face, Leg, Activity, Cry, Consolability scale and ease of treatment completion using a separate 5-point scale. Vital signs were recorded before and during sedation, until full recovery.

**Results:**

A significant difference was detected regarding children responsiveness during operative procedure in favour of DEX in comparison to MID and DEX/MID groups (*P* = 0.045). Within the DEX group, a significant difference was recorded regarding children responsiveness at optimum sedation and during the operative procedure (*P* = 0.04). Although, the analgesic effects of sedative drugs showed no statistically significant difference among the study groups (*P* = 0.20), the ease of treatment completion was statistically higher in the DEX than MID and DEX/MID groups (*P* = 0.03).

**Conclusion:**

Dexmedetomidine provides a moderate level of sedation, that allowed better patient cooperation, and easy completion of performed dental procedures.

**Supplementary Information:**

The online version contains supplementary material available at 10.1186/s12903-024-04992-2.

## Background

Dental anxiety and fear related behaviors are global problems among preschoolers. They present a significant challenge to pediatric dentists [1, 2]. According to American Academy of Pediatric Dentistry (AAPD), sedation is a form of advanced behavior guidance technique, indicated for uncooperative or fearful/anxious children [3]. This approach aims to control anxiety, minimize pain and enhance the patient’s safety, as well as to allow for the completion of the procedure successfully [3, 4]. A variety of sedative drugs have been commonly used in Dental Procedural Sedation (DPS) [5, 6]. No strong evidence was pooled toward the preference of any of the drugs or their combinations [7–9]. 

Midazolam (MID) is the most commonly used for dental procedural sedation as it is short-acting (average of 30 min work), anxiolytic, and sedative [10, 11]. In addition, MID is characterized by the ability to mediate a variable degree of amnesia [7]. According to Xiong et al. 2024 [12], 0.5 mg/kg of MID is an effective and safe dose for pediatric patients. However, sedation with MID sometimes produces deeper levels of sedation with accompanying respiratory depression, and induces paradoxical behavioural reactions specially in children [13]. In addition, if administered nasally it usually causes burning sensation, irritation, and lacrimation during instillation [14]. More recently, Dexmedetomidine (DEX) has been introduced clinically as a promising alternative in procedural sedation as compared to MID [15–17]. It is a sedative with anxiolytic, sympatholytic, and analgesic effects, with minimal depression of respiratory function [16, 18]. Dexmedetomidine (an alpha2 (α2)-adrenergic agonist) acts primarily on stimulation of parasympathetic outflow [16]. An important feature of DEX-based sedation is that it resembles natural sleep, so that the patient remains easily arousable [16]. Singh et al., proposed a dose of 5 μg/kg DEX as clinically suitable in terms of sedation level, analgesia, and overall success rate [19]. Moreover, it maintains spontaneous ventilation and preserves upper airway tone [17]. DEX is well tolerated, since it is a tasteless, colorless, and odorless agent that acts on the CNS [18]. However, sudden arousal in response to stimulation, especially sound, may be a disadvantage for dental applications [13]. Additionally, when compared with MID, it has a relatively slow onset of action [20, 21]. 

The combination of MID to DEX might be appropriate to obtain high-quality sedation and analgesia, allowing more components of the pain and anxiety response to be neutralized [10]. However, the use of drug combinations must be judicious in order to produce maximum results with minimum adverse outcomes. Therefore, the individual drug doses should be reduced by 20–50% when drugs are given in combination [10]. 

Although the use of DEX in dentistry has proven its effectiveness [22], more evidence concerning its use in pediatric population for dental procedures is needed [23]. To the authors’ best knowledge, scarce information has been reported on the effectiveness of DEX versus MID in children during dental treatment. The objective of the present study was to compare the efficacy of sedation with DEX with or without MID in terms of sedation level and ease of treatment completion in uncooperative children undergoing dental treatment. The proposed null hypothesis is that there is no difference between the sedative effect and ease of treatment completion between DEX, MID or a combination of both.

## Methods

### Study design

The study was a three-arm randomized controlled clinical trial with a parallel design. It was reported according to the Consolidated Standards of Reporting Trials (CONSORT) guidelines [24]. The PICO question was “Do patients aged 4–6 years undergoing dental treatment (P) sedated with DEX (I1), or DEX/MID (I2) in comparison to those sedated with MID (C) show better sedation, analgesia during local anaesthesia administration and ease of treatment completion (O)? The study protocol was approved by the research ethics committee at the Faculty of Dentistry, Alexandria University and registered on clinicaltrials.gov (Trial ID: NCT03827408) on (01/02/ 2019).

### Sample size estimation

The sample size was calculated to detect the medium sized standardized effect size (Cohen’s d), of 0.374 of the primary outcome (level of sedation based on Modified Observer’s Assessment of Alertness/Sedation Scale), as statistically significant with 80% power (β = 20%) and at a significance level of 95% (α = 0.05) [25]. The sample size was calculated using GPower version 3.1.9.2 [26]. based upon a study on the primary outcome from a previous study by Zanaty & Metainy [2]. A sample size of 24 per group (number of groups = 3) was the minimum required sample.

### Study sample

Seventy-two healthy children 4–6 years old (ASA I or II physical status) [27], attending the outpatient clinic of the Pediatric Dentistry and Dental Public Health Department, Faculty of Dentistry, Alexandria University were selected for this study. They required dental intervention under local anesthesia and showed negative behaviour according to Frankl behaviour rating scale (score 2) [28], where all basic behavior guidance techniques had failed in gaining child cooperation [7]. They were randomly assigned to one of the three arms of the study (1:1:1).

Those who had history of neurological or cognitive alterations or who were mouth breathers, were excluded [1]. Parents of children enrolled in the study were given a full explanation of the procedure, and a written consent was obtained.

### Randomization, and allocation concealment

A computer-generated list of random numbers was used to assign children randomly to one of the three arms. Allocation was performed by permuted block technique, where the list of allocation was generated prospectively using random allocation software where participants were allocated in blocks of 4. Each allocation was represented by a code (the serial of the participant in the study) and the group name. The allocation was sealed in opaque envelopes. The study was triple blind: The clinician, statistician and participants were blinded to the intervention group. However, the anaesthesiologist was aware of the allocation group.

### Grouping

Children enrolled in the study were randomly assigned to one of the three arms; Group I (*n* = 24): DEX group (IV Ampoules of Precedex 4mcg/ml, Hospira. Inc., Lake Forest, IL USA) received a nebulized solution of 5 μg/kg DEX [19]. Group II (*n* = 24): DEX/MID group received a nebulized solution of 3 μg/kg DEX followed by 0.3 mg/kg MID [10]. (IV Ampoules of Dormicum 15 mg/ 3 ml, Hoffman-La Rouche Ltd., Basel, Swithzerland). Group III (*n* = 24): MID group received a nebulized solution of 0.5 mg/kg MID [29]. All the drugs were administered through nebulization [30]. 

### Intra-examiner reliability

A pilot study was performed on15 patients who were not included in the study. Intra-examiner reliability of the clinician was tested by watching the videotapes of the 15 patients within 6 days intervals between the views. Kappa was equal 0.65, 0.83, 0.82 and for the sedation level, ease of treatment completion, and analgesic effect respectively with *p* value < 0.001* which indicated a moderate to good agreement.

### Pre-procedural visit

A full record that included demographic data, a brief medical history, airway evaluation [31], dental examination, and behavioural assessment was performed for the seventy- two patients enrolled in the study [28]. Pre-sedation fasting instructions according to AAPD guidelines were given [32]. Each child was then given an appointment and was scheduled to arrive at least one hour before the treatment procedure.

### Intervention

On the day of the procedural visit, the child accompanied by his/her parents was admitted to the operatory and ensured that he/she was fit for sedation by the anaesthesiologist. The child was asked to pick a closed envelope containing one of the regimens to be employed from among 72 white papers prepared and folded three times so as not to show its contents to assure random assignment. Baseline vital signs were recorded, including blood pressure (BP) using the digital wrist sphygmomanometer, heart rate (HR), and oxygen saturation (SaO_2_) via finger pulse oximeter ( Finger pulse oximeter, Granzia, Inc.) Body weight was recorded using a digital weight balance (Granzia BF 200 Balance Analysis, Granzia, Inc.) to determine the appropriate dose of the sedative drug and local anesthetic [33]. 

All the 3 sedative regimens in the current study were inhalational sedation; administered via nebulizer (PURE Nebulizer, Granzia, Inc.). The sedative drug according to the randomization plan was freshly prepared by the anesthesiologist from parenteral forms. To secure the blindness of the study, the proper designated drug dosages were made to an equal final volume by adding distilled water [34], then were divided equally into two identical syringes and labeled as; syringe 1 and syringe 2. In case of using a single sedative drug (Group I, III) the dosage was divided into two syringes. In case of combing two sedative drugs (Group II) each drug was loaded in a different syringe to avoid mixture during administration. Only the anesthesiologist was aware of the administered drugs. Before nebulization, tell, show, do technique was used to coach each child to breathe through the nose while keeping the mouth closed.

After administration of sedative drug, the children were allowed to stay in a quiet supine position on dental chair till reaching the calm or drowsy stage according to Wilton et al., [35] which is considered the optimum sedation state. Optimum sedation is when the child is in a sleep-like but easily arousable state and can comply with the therapeutic procedures [36, 37]. The time from drug administration until the optimum sedation state was prolonged for children sedated with DEX (17.08 ± 5.88 min) in comparison to MID (11.88 ± 5.48 min) and DEX/MID (8.33 ± 4.34 min). At this state, the sedation level of the child was assessed by the clinician and scored according to the Modified Observer’s Assessment of Alertness/Sedation Scale (MOAA/S) [38]. Then, A suitable sized mouth prop with a tongue retractor was placed, topical anesthetic gel (Benzocaine 30mL 20%, OPAHL™, DHARMA RESEARCH INC., USA) was applied followed by local anesthesia (Mepivacaine HCL 2% and levonordefrin1:20 000, Alexandria Co. For Pharmaceuticals & Chemical Industries, Egypt), according to the manufactures’ instructions [32]. During local anesthesia injection, the analgesic effect of each sedative drug was assessed using Face, Leg, Activity, Cry, Consolability scale “FLACC.” [39] A quadrant operative dental procedure was performed at which the sedation level was reassessed according to MOAA/S scale [38]. 

After completion of the dental procedure, the child was monitored for recovery. Postoperative care instructions concerning the sedation and dental procedure were given to the parents and the child was discharged according to the criteria of AAPD guidelines [38]. 

### Outcome assessment

The final outcome assessment was done through a video recorded. Each session was video recorded and the film was given a specific code equivalent to child’s serial number for assessment of outcomes:

I. Sedative effect of MID, DEX and their combination using MOAA/S scale [38].
II. Analgesic effect of the sedative drugs during local anesthesia injection using “FLACC.” [39].
III. The ease of treatment completion using separate 5-point scale [18].

### Statistical analysis

Normality was checked for all variables using descriptive statistics, plots, and Kolmogorov–Smirnov test of normality. Means and Standard Deviations (X ± SD) were calculated for all quantitative variables. Kruskal–Wallis Test and Wilcoxon signed rank test were used to compare the 3 groups, followed by post-hoc tests with multiple comparisons using Bonferroni adjustment for significant differences. Significance was set at *P* ≤ 0.05. Data was analysed using IBM SPSS statistical software (version 23).

## Results

**Fig. 1:**
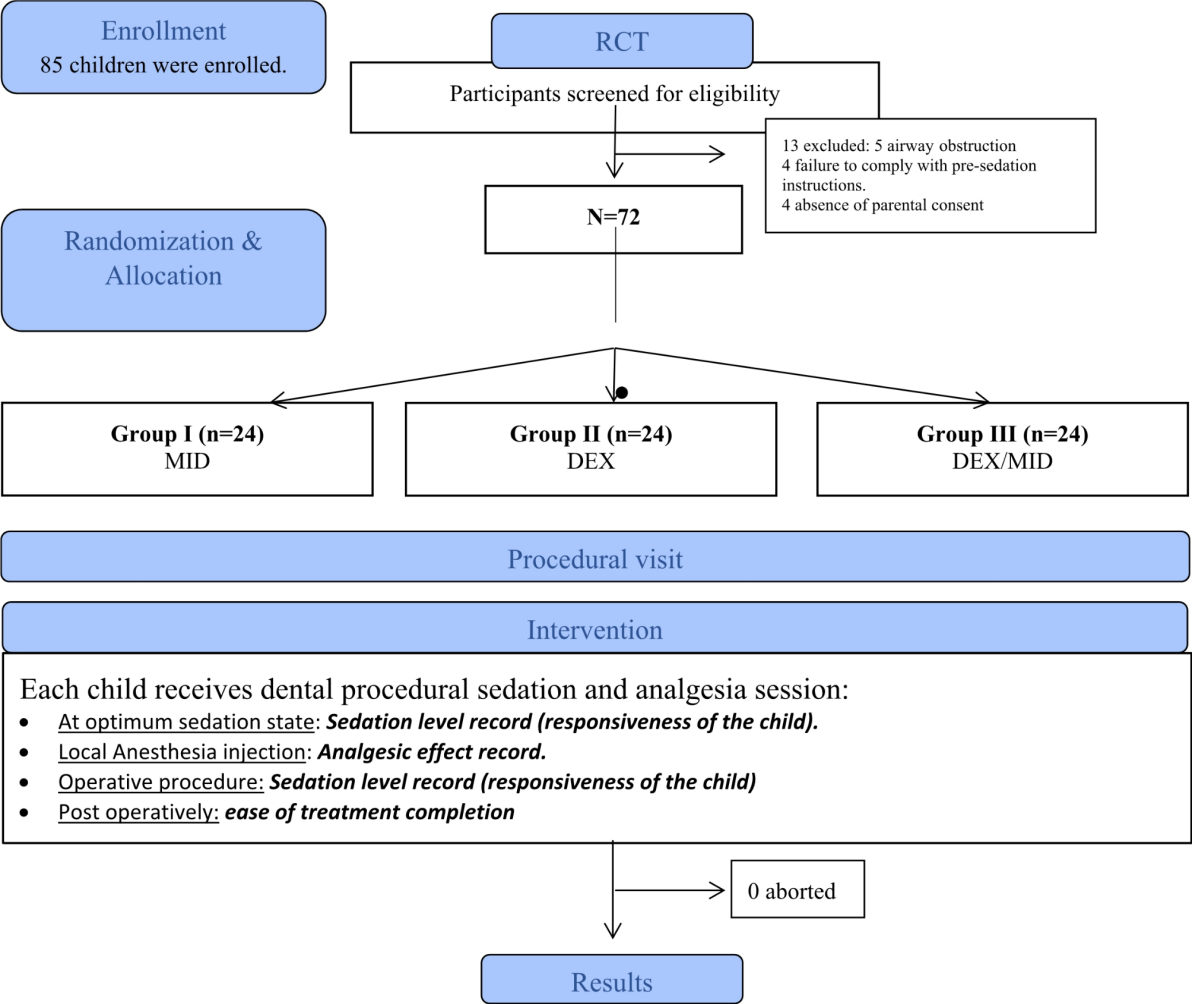
Research design flow chart according to CONSORT

Eighty-five children were screened for eligibility; seventy-two children who met the inclusion criteria were enrolled in the study. In the current study, recruitment of children extended for 3 months starting March 2020. None of the cases had been aborted. (Fig. 1 CONSORT Research design flow) chart. These included 44 males and 28 females, with no statistically significant difference between the three groups regarding age or sex (*P* = 0.06 and 0.47 respectively). The dental visits were also comparable in terms of type (restorations, pulpotomies and stainless-steel crowns, and/ or extractions)., number of procedures per quadrant in all groups, where each child received a quadrant dental treatment. (*P* = 0.13 and 0.23 respectively). As well, no statistical significant difference was detected between procedures performed neither in mandibular nor maxillary arch among all groups (*P* = 0.41). The safety of sedative drugs was assessed in terms of vital signs; blood pressure (BP), heart rate (HR), and oxygen saturation (SaO2). All vital signs were within normal range at baseline, mild bradycardia was detected with DEX but these effects were clinically insignificant, and no intervention was required. However, the oxygen saturation and blood pressure were unchanged during the whole sessions among all children.

### Sedation level assessment

No statistically significant difference was detected regarding the responsiveness of children using MOAA/S scale at optimum sedation among the three study groups (*P* = 0.21). However, a statistically significant difference was recorded during operative procedure between them (*P* = 0.04) (Table 1). According to post-hoc for multiple comparisons using Bonferroni adjustment a significant difference was detected between DEX and DEX/MID in favor of DEX group (*P* = 0.045).

A statistically significant difference was also detected among children of the DEX group between their responsiveness at optimum sedation and during the operative procedure (*P* = 0.04) (Table 1).

**Table 1:** Effect of sedative drugs on responsiveness at optimum sedation and during operative procedures

	Responsiveness score	DEX *n* (%)	DEX/MID *n* (%)	MID *n* (%)	Test value ^£^ *P* value
At optimum sedation	5	0 (0%)	0 (0%)	0 (0%)	3.14 0.21
	4	2 (8.3%)	5 (20.8%)	7 (29.2%)	
	3	11 (45.8%)	10 (41.7%)	14 (58.3%)	
	2	9 (37.5%)	9 (37.5%)	1 (4.2%)	
	1	0 (0%)	0 (0%)	2 (8.3%)	
During Operative Procedure	5	0 (0%)	0 (0%)	0 (0%)	3.45 0.04*
	4	4 (16.7%)	2 (8.3%)	2 (8.3%)	
	3	20 (83.3%)	15 (62.5%)	20 (83.3%)	
	2	2 (8.3%)	7 (29.2%)	2 (8.3%)	
	1	0 (0%)	0 (0%)	0 (0%)	
Test value^π^ *P* Value		-0.21 0.04*	-0.33 0.74	-0.37 0.71	

^£^: Kruskal - Wallis Test

^**π**^: Wilcoxon -signed rank test

*Statistically significant at *P* ≤ 0.05

### Analgesic effect assessment

No statistically significant difference was detected between components of FLACC regarding the analgesic effects of sedative drugs during local anesthesia injection among the study groups (*P* = 0.20). (Table 2)

**Table 2:** Analgesic effect of the three sedatives during local anesthesia injection using FLACC scale

SCALE	Mean ± SD			Test value ^£^ *P* value
	DEX	DEX/MID	MID	
**F = Face** (out of 2)	0.67 ± 0.82	0.50 ± 0.78	0.92 ± 0.78	4.01 0.14
**L = Leg** (out of 2)	0.63 ± 0.82	0.63 ± 0.88	0.88 ± 0.80	1.96 0.38
**A = Activity** (out of 2)	0.63 ± 0.88	0.58 ± 0.83	0.75 ± 0.85	0.67 0.72
**C = Cry** (out of 2)	0.92 ± 0.78	0.75 ± 0.90	1.25 ± 0.79	4.47 0.11
**C = Consolability** (out of 2)	0.92 ± 0.72	0.73 ± 0.96	1.25 ± 0.72	4.47 0.11
**FLACC** **(out of 10)**	3.75 ± 3.53	3.29 ± 4.03	4.80 ± 3.68	3.18 0.20

£: Kruskal - Wallis Test

*Statistically significant at *P* ≤ 0.05

### Ease of treatment completion

A statistically significant difference was detected between the MID group and DEX group (*P* = 0.03) using Bonferroni adjustment for multiple comparisons. Whereas 37.5% of children in DEX group showed excellent and quite behavior during treatment versus 8.3% of MID group. However, DEX/MID group did not show better scores regarding ease of treatment completion, with no statistically significant differences with either MID or DEX groups. (Table 3)

**Table 3:** Ease of treatment completion among study groups

Score	DEX ^b^ *n* (%)	DEX/MID ^a, b^ *n* (%)	MID ^a^ *n* (%)	Total *n* (%)
5	9 (37.5%)	9 (37.5%)	2 (8.3%)	20 (27.8%)
4	3 (12.5%)	5 (20.8%)	2 (8.3%)	10 (13.9%)
3	7 (29.5%)	1 (4.2%)	9 (37.5%)	17 (23.6%)
2	5 (20.8%)	9 (37.5%)	11 (45.8%)	25 (34.7%)
1	0	0	0	0
Test value^£^ *P* value	**6.81** 0.03*			

£: Kruskal - Wallis Test

*Statistically significant at *P* ≤ 0.05

^a, b^ Different letters denote statistically significant difference, but similar letters denote no statistically significant differences between groups using Bonferroni adjustment for multiple comparison

## Discussion

The current research aimed to compare the sedative effect of three procedural sedation regimens: Dexmedetomidine, Midazolam and their combination, evaluate their analgesic effect and assess the ease of treatment completion. Based upon the findings of the current study, the null hypothesis was partially rejected. The responsiveness of children who were sedated with DEX and MID were consistent in the range of moderate sedation at optimum sedation state. However, during the operative procedure, children who were sedated with DEX seemed more relaxed than the others. Children sedated with DEX can be easily aroused, but they return back to relaxed state resembling natural sleep [15, 16]. Similar to the current findings, Neville et al., [40] concluded that DEX was a better alternative to MID as an anxiolytic drug. However, Hiwarkar et al., [41] stated that both DEX and MID have equivalent efficiency when used for minor oral surgeries in adults. This controversy might be related to the difference in response to sedative drugs between children and adults.

When considering the combination of DEX and MID, children who were sedated with this regiment showed deep sedation which could be related to a notable synergism that is characterized by deeper level of sedation. However, the combination between DEX and MID did not increase the anxiolysis. The children sedated with DEX/MID were not easily arousable but showed some struggling behaviour. Our findings were in accordance with Togawa et al., [42] and Sago et al., [43] where combination of different sedative drugs caused deeper level of sedation. Although, no enough clinical studies have explained the synergism between DEX and MID in children, this hypnotic synergism has been shown in a rat model, and was characterized by a deeper level of CNS depression [44]. 

Although DEX is known for its marked analgesic effect, the current study showed no significant difference between DEX and neither DEX/MID nor MID. A closer look at the components of FLACC scale, showed that children sedated with DEX or DEX/MID were more relaxed, exercised less movement and less crying during local anesthetic injection than those sedated with MID despite the insignificant difference. DEX could be used as an adjuvant to local anesthetics to improve analgesic effect, and duration and reduce the need for postoperative analgesia. These findings were comparable with other studies that proved that DEX was associated with better analgesic properties compared to MID [13, 45]. The analgesic effect of DEX results from its sympatholytic action [15]. This fact is still controversial, since in clinical practice, the pain scores are reduced without increasing the analgesic component, but it does not exhibit additional analgesic benefit compared to MID postoperatively [46]. The present research seems to coincide with the findings of Barends et al. [15], in terms of sedation and analgesia.

Clinicians’ satisfaction as expressed by ease of treatment completion is a summative assessment of sedation success. The goal of the current study was to accomplish dental procedures in the easiest and most comfortable setting. During operative procedures, children sedated with DEX showed less struggling behavior and were easily calmed down. It could be thus postulated that DEX enhanced the children’s comfort and allowed completion of dental procedures successfully and easily. In accordance to these findings, several studies reported the use of DEX to be associated with greater clinician satisfaction and better patient cooperation than MID. Contrary to this fact, Cheung et al., reported that DEX and MID equally resulted in clinician satisfaction [46]. 

It has been reported that the combination of MID with other drugs was significantly less often associated with successful treatment completion [11]. This was evident in the current study, where children sedated with DEX/MID were not significantly easier to treat. This could be explained on the assumption that combining DEX and MID did not enhance the anxiolytic effects of either drug. The same finding was also observed in a study by Wakita et al., [13] who stated that the combination of DEX and MID during dental surgery was not associated with more intraoperative comfort. However, Sago et al. [43]. , reported a successful dental treatment after administration of a combination of DEX and low-dose MID for an uncooperative pediatric patient. They postulated that DEX and MID combination provides a helpful option for sedation in pediatric patients [43]. 

When DEX was compared to MID, the former provided a unique form of sedation in which children appeared relaxed, cooperative, and responsive when stimulated. Although, there have been reports of the synergistic effect when MID and DEX are used in combination [42, 43], there was no clear explanation of the type of synergism observed. One of the limitations of the present study was the hampered operator blindness, originally, the study was designed to ensure operator blinding, but due to the sleep induced by DEX, together with its slow onset, the drug administered was identified. Another limitation was the lack of assessment of the amnesic effect and the plasma level measurement of the sedative drugs, to evaluate their pharmacodynamic effect at different time intervals. Further investigations of dosages required to establish the optimal effect of DEX/MID are still needed in order to prevent arousal and maintain safe sedation during dental treatment in pediatric patients.

## Conclusion

Dental procedural sedation and analgesia is a valuable and reliable behavior guidance technique in children. Dexmedetomidine, Midazolam or their combination seem to be effective during moderate procedural sedation for children undergoing dental treatment. Dexmedetomidine appears to be more clinically reliable and safe in terms of sedation level, and ease of treatment completion. Despite of its insignificant analgesic effect during local anesthesia, its clinical performance exceeds either DEX/MID or MID.

## Electronic supplementary material

Below is the link to the electronic supplementary material.

Supplementary Material 1

## Data Availability

The database generated and analysed for the study are available in patient’s records in the Pediatric Dentistry and Dental Public Health Department, Faculty of Dentistry, Alexandria University. These data are not for publish and were used under the licence for the current study. Data are available from the correspondent author upon reasonable request with the permission of the Pediatric Dentistry and Dental Public Health Department, Faculty of Dentistry, Alexandria University.
