# Supplementary material for: Effect of Dexmedetomidine with or without Midazolam during procedural dental sedation in children: a randomized controlled clinical trial
Source: BMC Oral Health. 2024 Oct 26;24:1298. doi: 10.1186/s12903-024-04992-2 (PMC11520047; doi:10.1186/s12903-024-04992-2)
Supplement: Supplementary file 1 — Supplementary Material 1 [file 12903_2024_4992_MOESM1_ESM.docx]

**Supplemental files**

**Modified Observer’s Assessment of Alertness/Sedation Scale (MOAA/S) Scale:**

The Modified Observer’s Assessment of Alertness/Sedation Scale (MOAA/S) Scale, is derived from the original Observer's Assessment of Alertness/Sedation scale, where the observer rates only the patient’s responsiveness during sedation.

Responsiveness:

| 5 | Respond readily to normal tone |
| --- | --- |
| 4 | Lethargic response to normal tone |
| 3 | Respond only after loud / repeated calling |
| 2 | Responds only after mid prodding or shaking words |
| 1 | No response to mid prodding or shaking |

**‘Face, Legs, Activity, Cry, Consolability’ (FLACC):**

The Face, Leg, Activity, Cry,and Consolability scale is a measurement used to assess pain for children between the ages of 2 months and 7 years or individuals that are unable to communicate their pain. The scale is scored in a range of 0–10 with 0 representing no pain.

| 0: | Relaxed and comfortable |
| --- | --- |
| 1-3: | Mild discomfort |
| 4-6: | Moderate discomfort |
| 7-10: | Severe discomfort or pain or both |

The scale has five criteria, which are each assigned a score of 0, 1 or 2.

| **Criteria** | **Score 0** | **Score 1** | **Score 2** |
| --- | --- | --- | --- |
| **Face** | No particular expression or smile | Occasional grimace or frown, withdrawn, uninterested | Frequent to constant quivering chin, clenched jaw |
| **Legs** | Normal position or relaxed | Uneasy, restless, tense | Kicking, or legs drawn up |
| **Activity** | Lying quietly, normal position, moves easily | Squirming, shifting, back and forth, tense | Arched, rigid or jerking |
| **Cry** | No cry (awake or asleep) | Moans or whimpers; occasional complaint | Crying steadily, screams or sobs, frequent complaints |
| **Consolability** | Content, relaxed | Reassured by occasional touching, hugging or being talked to distractible | Difficult to console or comfort |

**The ease of treatment completion’ will be measured using separate five-point scales** **modified from “AAPD sedation record”.**

| 5 | Excellent Quiet and cooperative |
| --- | --- |
| 4 | Good Mild objections &/or whimpering but treatment not interrupted |
| 3 | Fair Crying with minimal disruption to treatment |
| 2 | Poor Struggling that interfered with operative procedures |
| 1 | Prohibitive Active resistance and crying; treatment cannot be rendered |

**Wilton et al sedation scale:**

| **Agitated** | Clinging to parent and/or crying. |
| --- | --- |
| **Alert** | Awake, but not clinging to parent, may whimper but not crying. |
| **Calm** | Sitting or lying comfortably with eyes spontaneously open |
| **Drowsy** | Sitting or lying comfortably with eyes spontaneously closing but responds to minor stimulation. |
| **Asleep** | Eyes closed, rousable but does not respond to minor stimulation. |

The table below shows different procedure applied in different arch per group

|  | | | **DEX** | | **DEX/MID** | **MID** | | **Test Value**  **P Value** |
| --- | --- | --- | --- | --- | --- | --- | --- | --- |
| **Type of treatment**  **(n = 24)** | **Restorations** | | 5 (20.8%) | | 4 (16.7%) | 4 (16.7%) | | F =0.24  0.91 |
|  | **Stainless steel crown** | | 0 (0%) | | 1 (4.16%) | 2 (8.33%) | | F =0.66  0.35 |
|  | **Pulpotomy /stainless steel crown** | | 24 (100%) | | 24 (100%) | 22 (91.67%) | | X2= 4.11  0.13 |
|  | **Extraction** | | 3 (12.5%) | | 5 (20.83%) | 4 (16.6%) | | F =1.90  0.74 |
| **Arch**  **(n = 24)** | | **Maxillary** | | 9 (37.5%) | 6 (25%) | | 5 (20.8%) | X^2^= 1.80  0.41 |
|  |  | **Mandibular** | | 15 (62.5%) | 18 (75%) | | 19 (79.2%) |  |
